# Prehospital transesophageal echocardiography versus conventional advanced life support in out-of-hospital cardiac arrest (PHTEE–OHCA) – a randomized controlled pilot study

**DOI:** 10.1186/s13054-025-05805-w

**Published:** 2026-01-02

**Authors:** Stephan Katzenschlager, Nikolai Kaltschmidt, Maximilian Dietrich, Mascha Fiedler-Kalenka, Sascha Klemm, Othmar Kofler, Stefan Mohr, Christoph Eisner, Christopher Neuhaus, Christoph Simon, Markus A. Weigand, Frank Weilbacher, Erik Popp

**Affiliations:** Medical Faculty Heidelberg, Department of Anesthesiology, Heidelberg University, Im Neuenheimer Feld 420, Heidelberg, 69120 Germany

**Keywords:** Out-of-hospital cardiac arrest, Transesophageal echocardiography, Advanced life support, Emergency medical service, extracorporeal cardiopulmonary resuscitation

## Abstract

**Background:**

Transesophageal echocardiography during out-of-hospital cardiac arrest can be performed during ongoing chest compressions and may improve resuscitation quality, but its prehospital use has not been systematically evaluated. To assess the feasibility, diagnostic yield, and impact of prehospital TEE on resuscitation metrics and advanced life support (ALS) interventions during OHCA.

**Methods:**

We conducted a randomized controlled trial in a physician-staffed two-tiered emergency medical service (EMS). Adults with ongoing non-traumatic OHCA were randomized 1:1 to standard ALS or ALS plus TEE. The primary endpoints were hands-off time and chest compression fraction (CCF) from EMS arrival to return of spontaneous circulation (ROSC) or resuscitation termination. Secondary endpoints included ROSC at hospital admission, survival to hospital discharge, neurological status at hospital discharge, and TEE findings. Analyses followed the intention-to-treat principle.

**Results:**

Of 249 screened patients, 35 were randomized and 32 analyzed (TEE *n* = 15; control *n* = 17). Median hands-off time was 4 s in both groups. Mean CCF was higher in the TEE group (96.2%) than the control group (91.6%), with a mean difference of 4.6% (95% confidence interval 2.5–6.7; *p* < 0.001). Sustained ROSC occurred in 40% (TEE) versus 71% (control; *p* = 0.083). The control group had an eCPR rate of 41%, compared to 20% in the TEE group. Using TEE, an incorrect area of maximal compression or inadequate depth was identified in 23% and 14%, respectively.

**Conclusion:**

Prehospital TEE during OHCA was feasible without negatively interfering with CPR metrics, and provided clinically relevant diagnostic information and procedural guidance, warranting further evaluation in larger trials.

**Trial registration:**

German Clinical Trials Register DRKS00028695 registered on 28 April 2022.

**Supplementary Information:**

The online version contains supplementary material available at 10.1186/s13054-025-05805-w.

## Introduction

Current European Resuscitation Council Advanced Life Support (ALS) guidelines recommend the use of transthoracic ultrasound to detect reversible causes of out-of-hospital cardiac arrest (OHCA) [1]. Pericardial tamponade and pneumothorax can be reliably diagnosed with ultrasound in patients with OHCA [2]. Although it is recommended to place the probe during ongoing chest compression in the presumed acoustic window [1], transthoracic echocardiography (TTE) carries the risk of prolonging chest compression pauses [3, 4]. As an alternative, transesophageal echocardiography (TEE) can be performed with the advantage of allowing image assessment during continuous chest compressions [5]. In-hospital usage of TEE for cardiac arrest patients has identified an area of maximal compression (AMC) over the aortic root of the left ventricular outflow tract (LVOT) in over half of the cases [6]. Similar findings have been demonstrated in a prehospital observational study [7]. Performing a TEE in the early stages of OHCA has the potential to improve patient outcomes by identifying reversible causes and improving the AMC compression to improve the low-flow state [6, 8, 9]. An analysis from a resuscitative TEE registry shows that the most common indication is evaluating patients with ongoing cardiac arrest, followed by post-cardiac arrest care and patients in shock [10].

In-hospital TEE was associated with shorter compression pauses compared to TTE [11]. The prehospital setting differs, with fewer personnel, no standardized space, and non-ideal patient positioning, leaving the impact and potential delay of prehospital TEE on chest compression pauses and recommended ALS interventions unclear. Prehospital TEE has the potential to change the management of OHCA patients in the early phase of cardiac arrest in standard ALS; further, the safety of extracorporeal cardiopulmonary resuscitation (eCPR) can be increased due to visualization of guidewires and the correct placement of the venous cannula [12].

This randomized controlled pilot study aimed to investigate potential interferences during chest compression pauses and ALS interventions that could diminish the overall quality of treatment. Further, we report on the changes in the AMC, possible causes of cardiac arrest, and guidance in eCPR cases.

## Methods

### Study design and setting

This pilot randomized controlled trial (RCT) was conducted within an emergency medical service (EMS) system serving 500,000 people. The two-tiered EMS includes ambulances staffed with a paramedic and technician, and physician response units dispatched for all OHCA cases. Five independent EMS agencies operate in the region, dispatched by a central center. These agencies have different procedures, especially regarding CPR feedback sensors. All units use Corpuls C3 defibrillators capable of manual rhythm analysis, defibrillation, and capnography.

Additionally, the Medical Intervention Car (MIC), consisting of two anesthesiologists and one cardiologist, responded to OHCAs who may be eligible for prehospital extracorporeal cardiopulmonary resuscitation (eCPR). The MIC carries equipment to perform prehospital TEE and eCPR on scene [13].

Before enrollment, the study was approved by the Ethics Committee of the Medical Faculty of Heidelberg University, Germany (protocol ID S-347/2021) and registered at the German Clinical Trials Register (number DRKS00028695) on April 28, 2022.

The study results are reported following the Consolidated Standards of Reporting Trials guidelines, with the recommended extension for pilot trials [14] (Supplement Checklists 1 and 2). The Heidelberg Surgical Foundation funded this study.

### Randomization

The principal investigator conducted randomization. Sequence allocation was performed using https://www.randomizer.org with a 1:1 ratio and a fixed block size of six. Identical sealed envelopes were carried in the MIC. A member of the MIC team opened the sealed envelope, allocating the patient to either the intervention group or the control group. The principal investigator was the only person with access to the allocation sequence.

### Patient inclusion

Participants were eligible for inclusion if they were ≥ 18 years of age and had ongoing OHCA at the time of the study team’s arrival. Patients with suspected traumatic OHCA were excluded. Further, patients with absolute contraindications for TEE according to the American Society of Echocardiography guidelines, for example, with known esophageal diseases, were excluded (Supplement Table 1) [15]. On scene, the eligibility of the patients was checked by one member of the MIC team.

Initial trial enrollment was approved as an emergency exemption for patients unable to give informed consent temporarily. For survivors until hospital admission, consent was sought from the patient, relatives, or legal proxy as soon as possible. Patients were included without further consent under an emergency exemption when CPR was stopped on scene.

### Treatment groups

Patients allocated to the standard group were treated according to the European Resuscitation Council ALS 2021 guidelines, which included the potential to perform TTE [1]. However, TTE was not mandatory in the standard group and was decided by the treating physician.

Patients allocated to the intervention group received TEE in addition to the standard of care. To receive TEE, patients had to be endotracheally intubated. A member of the MIC team performed prehospital TEE after arriving on the scene. TEE was performed following a structured approach previously described by the study group [16]. After the study inclusion criteria were fulfilled, the TEE-specific contraindications were checked. TEE was performed in all patients with ongoing cardiac arrest randomized to the intervention group. The indication for TEE was not based on clinical judgement. One member of the study team positions themselves at the patient’s head and prepares the TEE machine. The use of a bite block is mandatory in our setting. The endotracheal tube is secured with either a commercial device or a bandage; additionally, one member of the EMS team manually secures the tube for TEE insertion. After insertion, up to four standard views should be obtained as previously described [16].

The TEE assessment was performed using the GE Healthcare Venue Go™ device. All findings were communicated to the treatment team; therefore, medical personnel were not blinded to the intervention. All members had previous experience in TEE, either from cardiac anesthesiology or critical care echocardiography as part of their special training in intensive care medicine. Prehospital TEE was not the standard of care before the start of this study for OHCA.

Cross-over from the control to the intervention group was allowed if the patient received prehospital eCPR. This ensured the safe cannulation with visualization of the guidewires and venous cannula.

### Sample size

At the time of study planning, no previous prehospital data on TEE-guided resuscitation were available to estimate effect size or variance for CPR metrics or clinical outcomes. CPR metrics were not recorded in our EMS system before this trial. Therefore, a formal sample size calculation was not feasible. A convenience sample size of 30 patients (15 per group) was chosen, following the recommendations from the Institute of Medical Biometry and Informatics, Ruprecht-Karls University of Heidelberg. Patients were randomized and included until the sample size was achieved in both groups.

### Outcomes and data definition

The primary outcome was the difference in hands-off time and chest compression fraction between the two groups on an intention-to-treat basis. Hands-off time was defined as the pause in chest compression after the initiation of CPR, regardless of the nature of the pause. Chest compression fraction (CCF) was calculated as the ratio of chest compression time to total resuscitation time, with hands-off intervals measured from the first compression detected through changes in thoracic impedance until ROSC or termination of resuscitation [17]. CCF was calculated over the whole course of resuscitation. If the patient had any ROSC during the prehospital phase, the duration with spontaneous circulation was excluded from the calculation of the CCF.

The main secondary outcomes were patient-centered, including sustained ROSC at hospital admission, survival to hospital discharge, and survival to hospital discharge with a good neurological outcome. The neurological outcome was defined according to the cerebral performance category, where a score of 1 to 2 is considered a good neurological outcome, 3 to 4 a poor neurological outcome, and 5 indicates death [18]. Further secondary endpoints include complications associated with TEE, potential causes of OHCA, changes in end-tidal CO2 (etCO2), and eCPR guidance (Supplement Table 2). The TEE image quality was judged by the study team by interdisciplinary consensus, blinded to the cases, and categorized into ‘good’, ‘acceptable’, ‘poor’, and ‘not possible’. The TEE findings are reported in an as-treated analysis, including all available data. To analyze the effect of cross-overs, we performed a per-protocol analysis for the primary outcome.

### Data analysis

Analyses were performed using SPSS (Statistical Product and Services Solutions, Version 30, SPSS Inc., Chicago, IL, USA). Demographic and baseline clinical characteristics were presented as means with standard deviations (SD) for parametric data or medians with 25th and 75th percentiles (IQR) for non-parametric continuous data, and as counts and percentages for categorical variables. The primary endpoint was annotated using the corpuls.manager REVIEW (corpuls | GS Elektromedizinische Geräte G. Stemple GmbH, Kaufering, Germany) software with manual annotations of chest compression artefacts [19]. Data was available from the start of the monitoring until the patient was handed over to the hospital team. To assess the distribution and heterogeneity of the differences in hands-off time, descriptive statistics including measures of central tendency, dispersion, skewness, and kurtosis were calculated separately for each study group. Normality was evaluated using the Shapiro-Wilk test, and variance homogeneity was assessed with Levene’s test. CCF was normally distributed, and a Student’s t-test with 95% confidence intervals (CI) was used. However, there was a significant deviation from normality for the hands-off time in the control group. Consequently, the Mann-Whitney U test was used, and the effect size was calculated as the difference between pseudo medians with a nonparametric 95% CI using the Hodges-Lehmann method. For comparing repeated etCO2 measurements, we used a linear mixed-effects model with subject-specific random intercepts. For etCO2 comparisons, we analyzed data up to ten minutes before and after the study team’s arrival, depending on availability.

Models were estimated by restricted maximum likelihood for inference, with maximum likelihood for comparison. We report fixed effect estimates with 95% CIs. EtCO_2_ levels were compared across all cases and in the intervention group to test the effect of TEE. Due to the low sample size, this model was not adjusted for confounders.

A two-sided significance level of 5% was considered statistically significant. Categorical data were compared using the chi-squared or Fisher’s exact test. The protocol states that during eCPR cannulation, no rhythm analysis should be performed, and continuous chest compressions are recommended. This creates a bias in assessing CCF in eCPR patients. Therefore, a sensitivity analysis was conducted, calculating CCF up to the eCPR decision point and comparing it with the overall CCF. ECPR cases were included in the patient outcomes.

## Results

**Fig. 1:**
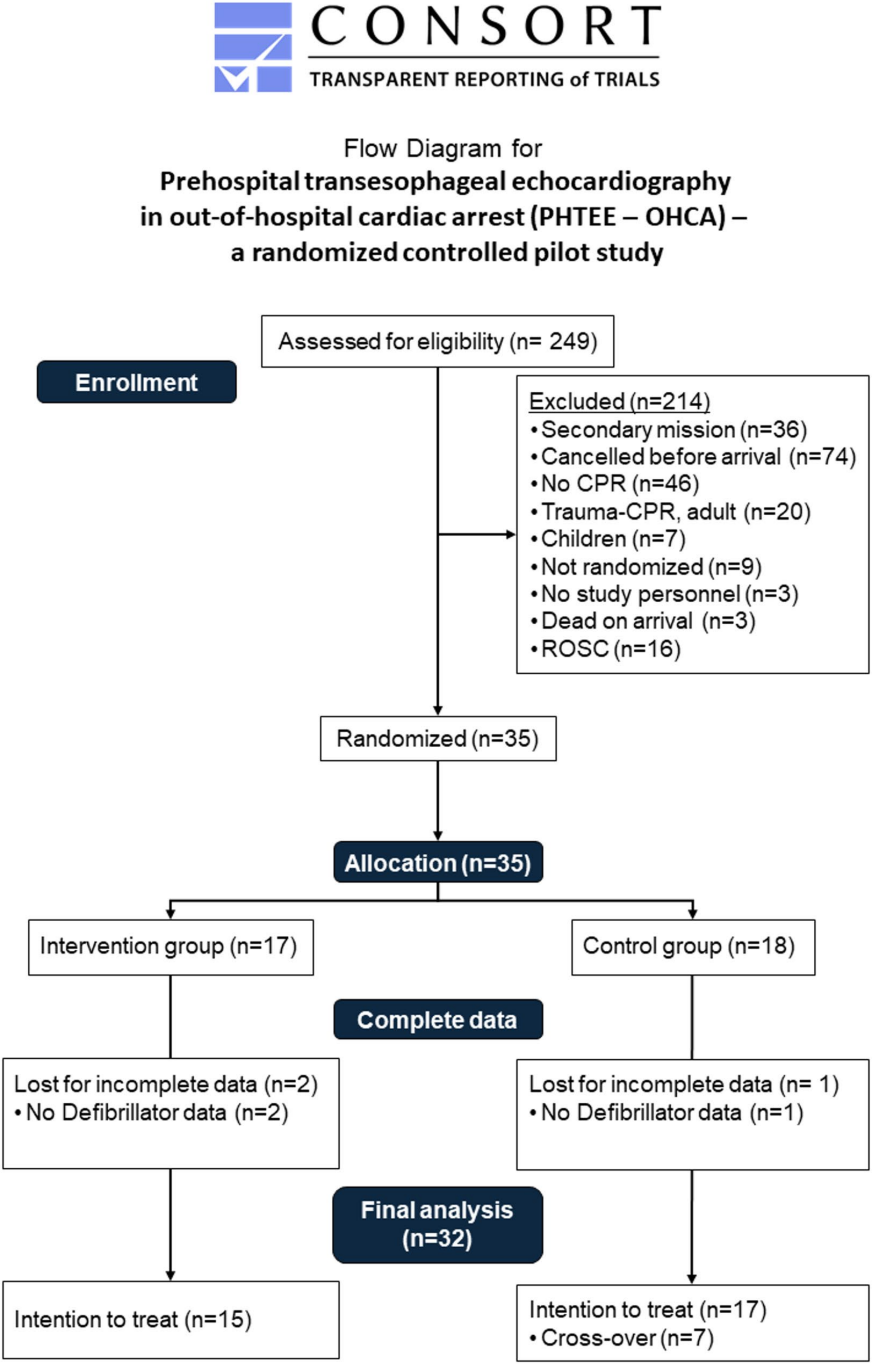
CONSORT 2025 Flow Chart of Patient Enrollment and Randomization [14]. This figure outlines the flow of patients through screening, randomization, and analysis. Of the 249 screened patients, 35 were randomized and 32 were included in the final analysis (TEE, *n* = 15; control, *n* = 17). Reasons for exclusion, cross-over to TEE in eCPR cases, and outcome ascertainment are displayed. CPR = cardiopulmonary resuscitation; ROSC = return of spontaneous circulation

Between July 27, 2022, and November 21, 2024, we screened 249 patients. Of those, 35 were randomized, 32 were included in the final analysis, with 15 analyzed in the intervention group and 17 in the control group (Fig. 1).

Participants randomized to the intervention group were older and had a higher proportion of females. In the control group, a higher rate of initial ventricular fibrillation and endotracheal intubation at the study team’s arrival was noted. Only the time from collapse to the arrival of the study team showed a significant difference (Table 1).

During the out-of-hospital treatment, all patients were endotracheally intubated.

TEE probe insertion and image acquisition were possible in all patients randomized to the TEE group.

**Table 1:** Baseline characteristics and treatment of the study population

	**TEE (n=15)**	**Standard ALS (n=17)**
Age, mean (SD)	68 (12)	58 (14)
Female, n (%)	5 (33)	3 (18)
Bystander CPR, n (%)	9 (60)	11 (65)
Time from collapse to study team arrival [min], mean (SD)*	22.0 (10.5)	30.5 (11)
Total CPR Duration [min], mean (SD)	49 (19)	52 (20)
Initial rhythm at EMS arrival, n (%) VF/pVT Asystole PEA	4 (27) 4 (27) 7 (47)	8 (47) 5 (29) 4 (24)
Endotracheal Intubated on arrival of study team, n (%)	11 (73)	16 (94)
Mechanical CPR, n (%)	4 (27)	8 (47)
Number of defibrillations, mean (SD)	2 (4)	4 (4)
Adrenalin dosage [mg], mean (SD)	4 (2)	5 (3)
Amiodarone dosage [mg], median (IQR)	0 (0–300)	300 (0 - 450)
Cross-Over, n (%)	0	7 (41)

This table summarizes demographic and clinical features of patients randomized to TEE (n=15) and control (n=17). Groups statistically differed only in time from collapse to study team arrival (*p=0.042).

SD = standard deviation; IQR = interquartile range; OHCA = out-of-hospital cardiac arrest; CPR = cardiopulmonary resuscitation; VF = ventricular fibrillation; pVT = pulseless ventricular tachycardia; PEA = pulseless electrical activity

### Primary Endpoint – CPR metrics

**Fig. 2:**
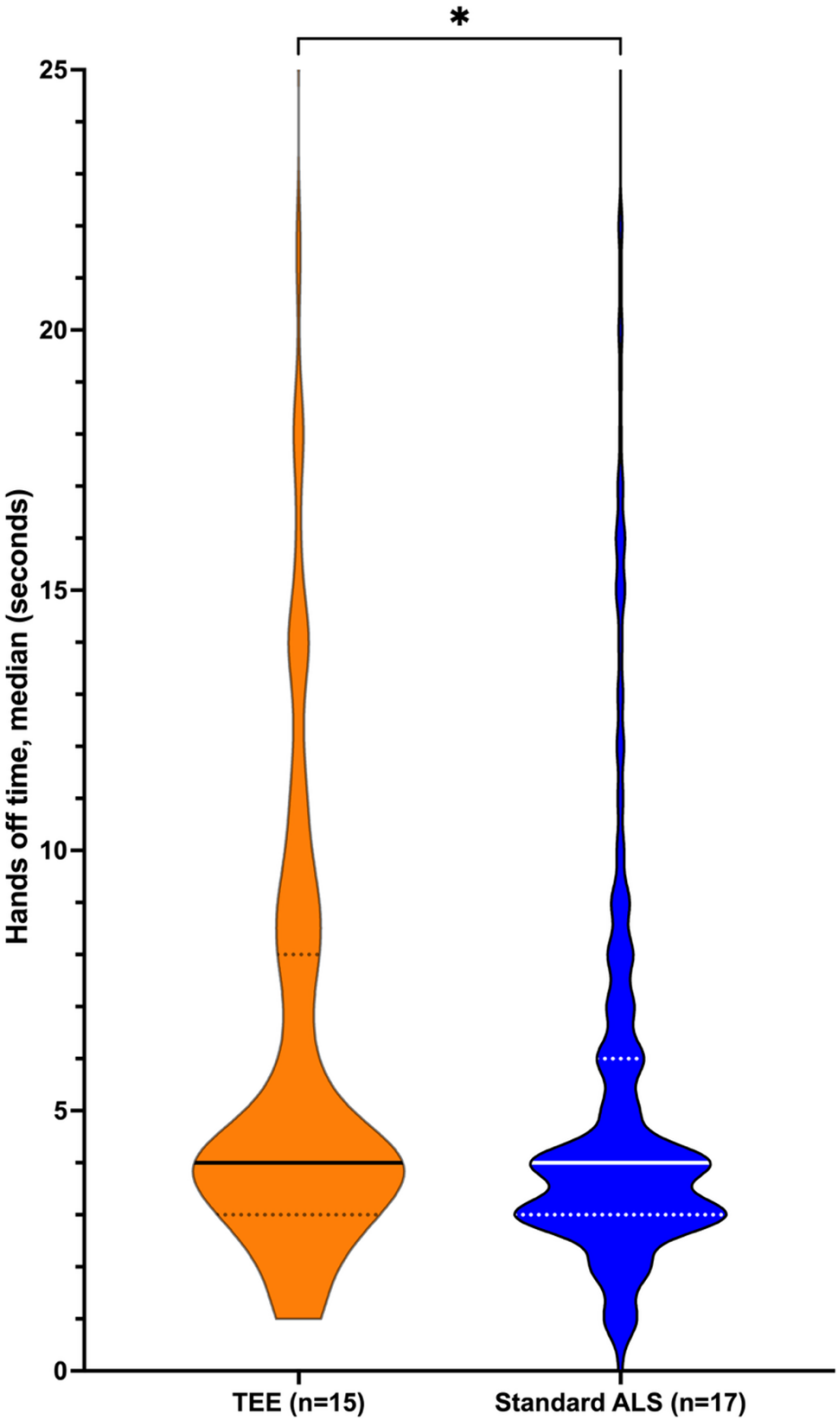
Distribution of Hands-Off Times During CPR: This Violin plot compares the distribution of hands-off times between the TEE and control groups. The median duration of each pause was 4 s in both arms (Difference 0 s, 95% CI 0–1). The distribution demonstrates minimal outliers in the TEE group, showing no adverse effect on chest compression interruptions. Outliers above 25 s are not visualized here to improve clarity. There were six outliers in the TEE group (Range 26–35 s) and 12 in the control group (Range 26–284 s)

The median (IQR) duration of each hands-off time was 4 (3–8) seconds in the TEE and 4 (3–6) seconds in the control group. This resulted in a difference of 0 s (95% CI 0 to 1) (Fig. 2). During TEE insertion, no pauses were recorded. There was no statistical difference in the rate of pauses > 10 s between the TEE and control groups (17% and 12%, *p* = 0.105, respectively). The median durations of pauses > 10 s were also comparable between the groups (TEE: 15 [IQR 13–21.25]; Control: 16.5 [IQR 14–28.25]; *p* = 0.206).

Median perishock pauses were similar, with 2 s (IQR 1–4) in the TEE group and 2 s (IQR 1–4.75) in the control group (*p* = 0.661).

Overall, CCF was 93.7% (SD 3.7). In the TEE and control groups, the CCFs were 96.2% (SD 2.4) and 91.6% (SD 3.3), respectively. This resulted in a significant mean difference of 4.6% (95% CI 2.5 to 6.7) in favor of the TEE group.

The per-protocol analyses consisted of 15 cases in the TEE group and 10 in the control group.

The median hands-off times were 4 s (IQR 3 to 8) in the TEE group and 4 s (IQR 3 to 6) in the control group (*p* = 0.288). The rates of hands-off times > 10 s did not differ between the TEE and control group (17% vs. 10%; *p* = 0.069). Analyzing these rates before and after the study team arrived showed an increase that was not statistically significant (Supplement Table 4).

Overall, CCF was lower in the control group than in the TEE group (90.7% [SD 3.3]) vs. 95.6% [SD 2.2]; *p* = 0.001).

### Secondary endpoints

ROSC was achieved in 23% and 20% in the control and TEE groups, respectively. eCPR was performed more often in the control group (47%) than in the TEE group (20%). Hospital discharge survival was 18% in the control group compared to 7% in the TEE group. A good neurological outcome was observed in 12% of the control group and 7% of the TEE group (Table 2).

**Table 2:** Main secondary outcomes of the study population

	**TEE (n=15)**	**Standard ALS (n=17)**	**p-Value**
Status at hospital admission, n (%) - Sustained ROSC - eCPR	3 (20) 3 (20)	4 (23) 8 (47)	0.809 0.108
Survival to hospital discharge, n (%)	1 (7)	3 (18)	0.422
Survival with CPC 1–2 at 30 days, n (%)	1 (7)	2 (12)	0.483

Patient-centered outcomes did not differ between the two study groups. A higher rate of eCPR cases was noted in the control group.

ROSC = return of spontaneous circulation; CPC = cerebral performance category; eCPR = extracorporeal cardiopulmonary resuscitation

In the as-treated analysis of TEE findings, no complications were noted. In 5/22 (23%) cases, the initial AMC was not over the left ventricle (Supplement Video 1) and had to be corrected (Supplement Video 2). Additionally, inadequate depth was recorded in three cases. After correction, AMC was over the LV in all cases and of adequate depth. In four patients, pericardial effusion was detected, three of whom were classified as having pericardial tamponade based on echocardiography findings (Supplement Video 3). Two of these tamponades resulted from an aortic dissection, while one was presumed to be a complication following an acute myocardial infarction (Table 3).

**Table 3:** Prehospital TEE findings across both groups (as-treated analysis)

	**TEE (n=22)**
Initial AMC over, n (%) - RV - LVOT - LV, adequate depth - LV, inadequate depth	3 (14) 2 (9) 14 (63) 3 (14)
Pericardial effusion, n (%) - Tamponade (% of Pericardial effusion)	5 (23) 3 (60)
Aortic dissection, n (%)	2 (9)
Complications, n (%)	0 (0)

This table presents TEE-derived diagnostic data from 22 patients who underwent imaging. More than one-third of the cases had incorrect areas of maximal compression or insufficient compression depth. Pericardial effusion with tamponade was observed in a significant number of patients. No TEE-related complications were reported.

AMC = Area of maximal compression; RV = right ventricle; LVOT = left ventricular outflow tract; LV = left ventricle

**Fig. 3:**
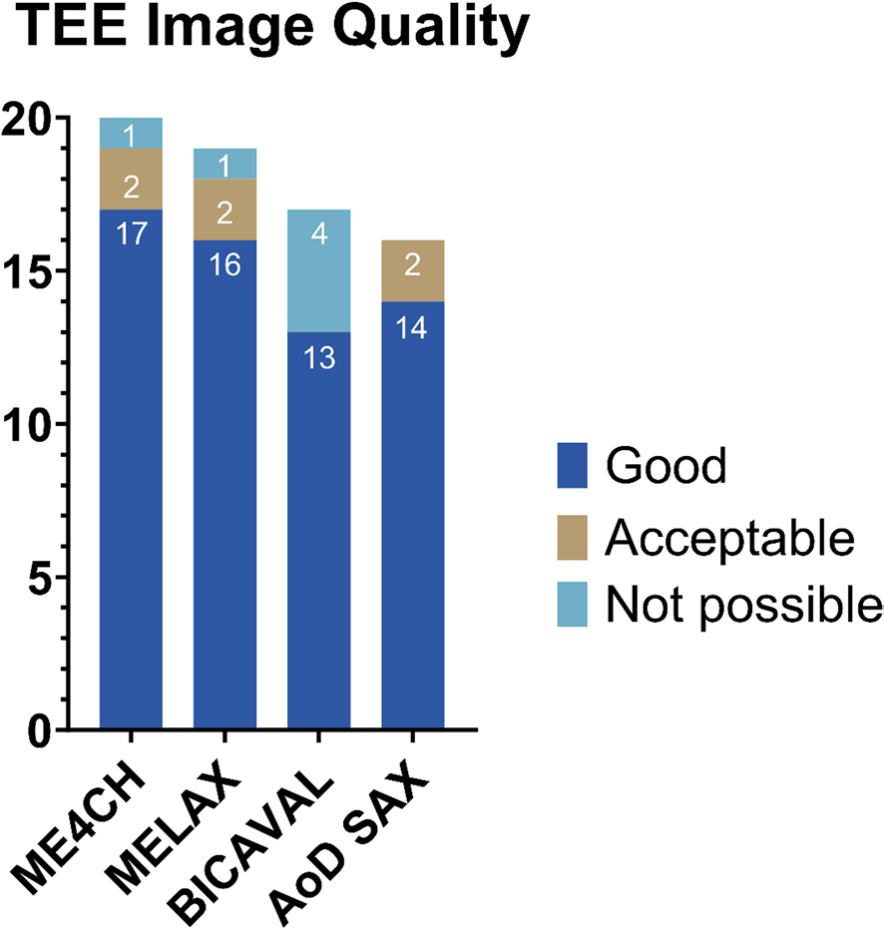
Image Quality of Prehospital TEE Views This bar chart summarizes image quality ratings for different TEE views in the as-treated population (*n* = 22). Most views were graded as “good”, particularly the mid-esophageal four-chamber. At least one diagnostic view was obtained in all patients. This underscores the technical feasibility and high yield of diagnostic imaging during ongoing CPR. ME4CH = Mid-esophageal four-chamber; MELAX = Mid-esophageal long axis; AoD SAX = Mid-esophageal Descending aortic short-axis

In the TEE group, the image quality was good in 83% of the cases, while the mid-esophageal four-chamber view had the highest rate of good image quality (Fig. 3). It was possible to obtain at least one view in all cases. In two instances, chest compressions had to be paused to acquire the mid-esophageal descending aorta, as it could not be visualized during chest compressions. In all eCPR cases, the visualization of both guidewires and the venous cannula was possible.

The mean etCO2 level in the control group was 24mmHg (SD 15), compared to 28mmHg (SD 21) in the TEE group. In the mixed-effects model with random intercepts, repeated etCO2 measurements before and after study team arrival showed an increase of 4mmHg (95% CI, 2 to 6; *p* < 0.001). In the TEE cohort, mean etCO_2_ levels before and after study team arrival were 24mmHg (SD 17) and 30mmHg (SD 27), respectively. In the mixed effect model, the etCO_2_ values were significantly higher in the TEE by 7mmHg (95% CI 4 to 10; *p* < 0.001).

### Sensitivity analysis CCF

Analyzing all cases until the timepoint where the decision to perform prehospital eCPR was made resulted in an overall CCF of 93.4% (SD 3.7), with a significant difference in favor of the TEE cohort (*p* < 0.001) (Supplement Table 3). Across both study groups, the CCF differed significantly before and after the study team arrived, with 92.3% (SD 5.3) compared to 95.7% (SD 3.3) (*p* = 0.01), respectively.

## Discussion

This study reports on the first randomized controlled trial on prehospital TEE in OHCA.

### Impact on CPR metrics and etCO_2_

In the TEE group, the mean CCF was significantly higher, with a mean difference of 4.6% (95% CI 2.5 to 6.7; *p* < 0.001). While observational studies have demonstrated feasibility [7, 8], this study corroborates this and adds that TEE does not lower chest compression fraction, prolong hands-off times, or interfere with standard ALS interventions. Our sensitivity analyses also demonstrate an increase in CCF after the study team’s arrival in both study arms. Although there was no statistically significant difference, the increased rate in hands-off times > 10 s in the per-protocol analysis should caution further systems when implementing prehospital TEE. Keeping this in mind, TEE can be performed, as insertion and rhythm analyses do not affect CPR metrics. CCF is known to be influenced by ventilation pauses, the total resuscitation durations, and team size [20]. With six to nine prehospital providers (three to four physicians, three to five paramedics) on scene, this study had a team size greater than usual OHCA settings. Having those resources potentially increases CPR metrics, as all ALS tasks could be done in parallel. In this study, five patients were not intubated when the study team arrived, potentially increasing hands-off time not attributable to TEE.

In the study by Cheskes et al., a higher CCF was not associated with improved outcome in patients with an initial shockable rhythm [21]. This can be attributed to the success of early defibrillation. However, Vaillancourt et al. found that, in patients with OHCA and non-shockable rhythm, a CCF of > 80 was associated with a higher adjusted odds ratio for ROSC [22]. In one quarter of the patients in the TEE group, intubation was performed after the study team arrived. This also prolonged the time until TEE insertion. Unfortunately, we have not recorded airway characteristics in this study.

Additional CPR quality parameters of interest include the depth and recoil of chest compressions. This was not recorded in this study; however, inadequate depth was noted in two patients using TEE. In those cases, the AMC was over the left ventricle, but compressions were initially poor and improved after verbal and visual feedback was provided to the EMS team. An observational study demonstrated that AMC correction increased the ability to palpate a brachial pulse from 16% to 77% of cases [7].

The higher etCO_2_ values observed in the TEE cohort may indicate more effective compressions, which is in line with experimental data [9, 23].

### Diagnostic yield and procedural guidance

In-hospital TEE has been shown to influence the management of cardiac arrest patients in 67% to 97% of cases concerning vasopressor use, AMC repositioning, and fluid boluses [6, 24, 25]. In this study, 23% of the AMC were incorrect at the initial evaluation, and an additional 14% had inadequate depth. However, the rate of incorrect AMC was lower compared to the study by Kruit et al. [7]; this proportion is comparable to those seen during in-hospital evaluations [6]. It should be acknowledged that there is currently no consensus on the assessment of the AMC. Aortic valve movement can be seen as a proxy for blood flow over the LVOT into the ascending aorta. In this study, we considered a correct AMC when (a) the left ventricle was adequately compressed in the mid-esophageal 4-chamber and mid-esophageal long-axis views, and (b) aortic valve opening was seen in the mid-esophageal long-axis view. If left ventricle compressions were deemed too shallow, EMS providers were instructed to increase the depth of chest compressions. When a compression of the left ventricular outflow tract was noted, the hand position of the person performing chest compressions was moved caudally under direct TEE guidance.

### Team size and systems considerations

In this study, at least two additional experienced ALS providers were present at the scene, which could influence the outcome of OHCA [26, 27]. The study team had no previous experience in prehospital TEE before the start of the study. The high number of providers on scene impacts the generalizability of this study. In low-resource settings, TEE might not be feasible due to the lack of human resources. Although this did not occur in this study, TEE devices may not work in specific temperature ranges. In systems with fewer providers, these findings may not be applicable, as they require one person to perform the TEE during cardiac arrest. Performing TEE should be seen as an additional tool when ALS is established.

Although the effect of TEE on survival remains unknown, it is plausible that early guidance of high-quality [5], addressing reversible causes, and enhancing the safety of invasive procedures like eCPR will be advantageous. Cannula malposition rates are approximately 5% across different centers [28, 29]. Due to patient safety factors for eCPR cannulation, crossover was allowed in this interventional trial. However, this paid off, as guidewires and venous cannula were visualized in all cases, and no malposition was recorded.

### Clinical utility of identifying causes

In total, five pericardial effusions were detected with TEE. Of those, three were hemodynamically relevant, with two due to an aortic dissection and one presumably because of a myocardial infarction. Hemodynamically relevant pericardial effusions were judged based on the size and right ventricular compression. In cases with aortic dissection, resuscitation efforts were immediately ceased, and the irreversible cause was communicated to the team and relatives. Although not quantified, the EMS service considered this information very helpful during debriefing. The study by Krammel et al. showed a median duration from arrival of the study team until a TEE image was acquired of five minutes [8]. In our study, this time is not routinely recorded and may be skewed due to the dual usage of the ultrasound machine for percutaneous vascular access with a linear probe.

### Outlook

Future studies should investigate the hemodynamic effects of prehospital TEE and its subsequent influence on immediate patient outcomes. To expand TEE accessibility to a wider patient population, training curricula should be assessed alongside in-field experience and reliability.

### Limitations

This study has several limitations. (A) Due to the pilot nature, the small sample size decreases the certainty of the effect sizes. (B) This was a single-center study with an additional team on scene; therefore, the generalizability is limited. As the additional team was on scene for all cases, this reduced the Hawthorne effect [30] in one group. However, the lack of blinding, combined with crossover, suggests a clinician bias towards using TEE. (C) Performing prehospital TEE requires additional equipment and expertise [31], limiting its applicability to other EMS systems. (D) The use of eCPR is a major confounder for survival to hospital admission. However, there was no significant difference in survival outcomes. This can be due to the numerically higher rate of bystander CPR and younger age in the control group. This pilot study was not powered to assess any outcome differences.

## Conclusion

In this RCT on prehospital TEE, CPR metrics are not impaired, and etCO2 is significantly higher using TEE. Further studies on the hemodynamic impact of early AMC correction are necessary.

## Supplementary Information

Supplementary Material 1

Supplementary Material 2

Supplementary Material 3

Supplementary Material 4

Supplementary Material 5

## Data Availability

The anonymized data set is available from the corresponding authors upon reasonable request.

## Abbreviations

ALS: Advanced Life Support
OHCA: Out-of-hospital cardiac arrest
TTE: Transthoracic echocardiography
TEE: Transesophageal echocardiography
AMC: Area of Maximal Compression
LVOT: Left Ventricular Outflow Tract
RCT: Randomized controlled trial
EMS: Emergency medical service
eCPR: Extracorporeal cardiopulmonary resuscitation
CCF: Chest compression fraction
