# Supplementary material for: Prehospital transesophageal echocardiography versus conventional advanced life support in out-of-hospital cardiac arrest (PHTEE–OHCA) – a randomized controlled pilot study
Source: Crit Care. 2026 Jan 2;30:45. doi: 10.1186/s13054-025-05805-w (PMC12849066; doi:10.1186/s13054-025-05805-w)
Supplement: Supplementary file 4 — Supplementary Material 4 [file 13054_2025_5805_MOESM4_ESM.docx]

**Supplementary Material to the Manuscript**

**Prehospital transesophageal echocardiography versus conventional advanced life support in out-of-hospital cardiac arrest (PHTEE – OHCA) – a randomized controlled pilot study**

Stephan Katzenschlager^a*^, Nikolai Kaltschmidt^a^, Maximilian Dietrich^a^, Mascha Fiedler-Kalenka^a^, Sascha Klemm^a^, Othmar Kofler^a^, Stefan Mohr^a^, Christoph Eisner^a^, Christopher Neuhaus^a^, Christoph Simon^a^, Markus A. Weigand^a^, Frank Weilbacher^a^, Erik Popp^a^

A Heidelberg University, Medical Faculty Heidelberg, Department of Anesthesiology, Heidelberg, Germany

* Address for correspondence:

Stephan Katzenschlager, MD

Heidelberg University, Medical Faculty Heidelberg, Department of Anesthesiology, Heidelberg, Germany

Im Neuenheimer Feld 420, 69120 Heidelberg, Germany

stephan.katzenschlager@med.uni-heidelberg.de

+4962215639683

Table of Contents

[Supplement Checklist 1 – CONSORT 3](#_Toc212377835)

[Supplement Checklist 2 – CONSORT Extension for Pilot Trials 6](#_Toc212377836)

[Supplement Table 1 – Inclusion and Exclusion Criteria 10](#_Toc212377837)

[Supplement Table 2 – Primary and secondary endpoints 11](#_Toc212377838)

[Supplement Table 3 – Sensitivity analysis CCF before decision to perform eCPR 12](#_Toc212377839)

[Supplement Table 4 – Rates of Hands off times >10s before and after the study team arrives 13](#_Toc212377840)

# Supplement Checklist 1 – CONSORT

| Section/topic | No | CONSORT 2025 checklist item description | Reported on page no. |
| --- | --- | --- | --- |
| **Title and abstract** | | |  |
| Title and structured abstract | 1a | Identification as a randomised trial | 2 / Abstract |
|  | 1b | Structured summary of the trial design, methods, results, and conclusions | 2 /Abstract |
| **Open science** | | |  |
| Trial registration | 2 | Name of trial registry, identifying number (with URL) and date of registration | 2 / below Abstract |
| Protocol and statistical analysis plan | 3 | Where the trial protocol and statistical analysis plan can be accessed | N/A |
| Data sharing | 4 | Where and how the individual de-identified participant data (including data dictionary), statistical code and any other materials can be accessed | 17 / Data sharing |
| Funding and conflicts of interest | 5a | Sources of funding and other support (eg, supply of drugs), and role of funders in the design, conduct, analysis and reporting of the trial | 17 / Funding sources |
|  | 5b | Financial and other conflicts of interest of the manuscript authors | 17 / Conflict of interest |
| **Introduction** | | |  |
| Background and rationale | 6 | Scientific background and rationale | 3 / Introduction |
| Objectives | 7 | Specific objectives related to benefits and harms | 3 / Introduction |
| **Methods** | | |  |
| Patient and public involvement | 8 | Details of patient or public involvement in the design, conduct and reporting of the trial | N/A |
| Trial design | 9 | Description of trial design including type of trial (eg, parallel group, crossover), allocation ratio, and framework (eg, superiority, equivalence, non-inferiority, exploratory) | 4 / Study design, Randomization |
| Changes to trial protocol | 10 | Important changes to the trial after it commenced including any outcomes or analyses that were not prespecified, with reason | N/A |
| Trial setting | 11 | Settings (eg, community, hospital) and locations (eg, countries, sites) where the trial was conducted | 4 / Study design and setting |
| Eligibility criteria | 12a | Eligibility criteria for participants | 5 / Patient inclusion  Supplement Table 1 |
|  | 12b | If applicable, eligibility criteria for sites and for individuals delivering the interventions (eg, surgeons, physiotherapists) | N/A |
| Intervention and comparator | 13 | Intervention and comparator with sufficient details to allow replication. If relevant, where additional materials describing the intervention and comparator (eg, intervention manual) can be accessed | 5, 6 / Treatment groups |
| Outcomes | 14 | Prespecified primary and secondary outcomes, including the specific measurement variable (eg, systolic blood pressure), analysis metric (eg, change from baseline, final value, time to event), method of aggregation (eg, median, proportion), and time point for each outcome | 6 / Outcomes and data definition  Supplement Table 2 |
| Harms | 15 | How harms were defined and assessed (eg, systematically, non-systematically) | 6 / Outcomes and data definition  Supplement Table 2 |
| Sample size | 16a | How sample size was determined, including all assumptions supporting the sample size calculation | 6 / Sample Size |
|  | 16b | Explanation of any interim analyses and stopping guidelines | N/A |
| Randomisation: |  |  |  |
| Sequence generation | 17a | Who generated the random allocation sequence and the method used | 4 / Randomization |
|  | 17b | Type of randomisation and details of any restriction (eg, stratification, blocking and block size) | 4 / Randomization |
| Allocation concealment mechanism | 18 | Mechanism used to implement the random allocation sequence (eg, central computer/telephone; sequentially numbered, opaque, sealed containers), describing any steps to conceal the sequence until interventions were assigned | 4 / Randomization |
| Implementation | 19 | Whether the personnel who enrolled and those who assigned participants to the interventions had access to the random allocation sequence | 4 / Randomization |
| Blinding | 20a | Who was blinded after assignment to interventions (eg, participants, care providers, outcome assessors, data analysts) | 5 / Treatment groups |
|  | 20b | If blinded, how blinding was achieved and description of the similarity of interventions | 5 / Treatment groups |
| Statistical methods | 21a | Statistical methods used to compare groups for primary and secondary outcomes, including harms | 7 / Data analysis |
|  | 21b | Definition of who is included in each analysis (eg, all randomised participants), and in which group | 7 / Data analysis |
|  | 21c | How missing data were handled in the analysis | 7 / Data analysis |
|  | 21d | Methods for any additional analyses (eg, subgroup and sensitivity analyses), distinguishing prespecified from post hoc | 7 / Data analysis |
| **Results** | | |  |
| Participant flow, including flow diagram | 22a | For each group, the numbers of participants who were randomly assigned, received intended intervention, and were analysed for the primary outcome | Figure 1 |
|  | 22b | For each group, losses and exclusions after randomisation, together with reasons | Figure 1 |
| Recruitment | 23a | Dates defining the periods of recruitment and follow-up for outcomes of benefits and harms | 8 / Results |
|  | 23b | If relevant, why the trial ended or was stopped | N/A |
| Intervention and comparator delivery | 24a | Intervention and comparator as they were actually administered (eg, where appropriate, who delivered the intervention/comparator, how participants adhered, whether they were delivered as intended (fidelity)) | Figure 1 |
|  | 24b | Concomitant care received during the trial for each group | 8 / Results |
| Baseline data | 25 | A table showing baseline demographic and clinical characteristics for each group | Table 1 |
| Numbers analysed,  outcomes and estimation | 26 | For each primary and secondary outcome, by group:  ● the number of participants included in the analysis  ● the number of participants with available data at the outcome time point  ● result for each group, and the estimated effect size and its precision (such as 95% confidence interval)  ● for binary outcomes, presentation of both absolute and relative effect size | 10 / Primary Endpoints  Figure 2  11 / Secondary Endpoints  Table 2  Table 3  Figure 3 |
| Harms | 27 | All harms or unintended events in each group | Table 3 |
| Ancillary analyses | 28 | Any other analyses performed, including subgroup and sensitivity analyses, distinguishing pre-specified from post hoc | 14 / Sensitivity analysis CCF  Supplement Table 3 |
| **Discussion** | | |  |
| Interpretation | 29 | Interpretation consistent with results, balancing benefits and harms, and considering other relevant evidence | 14, 15 |
| Limitations | 30 | Trial limitations, addressing sources of potential bias, imprecision, generalisability, and, if relevant, multiplicity of analyses | 16 |

# Supplement Checklist 2 – CONSORT Extension for Pilot Trials

| Section/Topic | Item No | Checklist item | Reported on page No |
| --- | --- | --- | --- |
| Title and abstract | | | |
|  | 1a | Identification as a pilot or feasibility randomised trial in the title | Title |
|  | 1b | Structured summary of pilot trial design, methods, results, and conclusions (for specific guidance see CONSORT abstract extension for pilot trials) | 2 / Abstract |
| Introduction | | | |
| Background and objectives | 2a | Scientific background and explanation of rationale for future definitive trial, and reasons for randomised pilot trial | 3 / Introduction |
|  | 2b | Specific objectives or research questions for pilot trial | 3 / Introduction |
| Methods | | | |
| Trial design | 3a | Description of pilot trial design (such as parallel, factorial) including allocation ratio | 4 / Methods |
|  | 3b | Important changes to methods after pilot trial commencement (such as eligibility criteria), with reasons | N/A |
| Participants | 4a | Eligibility criteria for participants | 5 / Patient inclusion |
|  | 4b | Settings and locations where the data were collected | 4 / Study design and setting |
|  | 4c | How participants were identified and consented | 5 / Patient inclusion |
| Interventions | 5 | The interventions for each group with sufficient details to allow replication, including how and when they were actually administered | 5 / Treatment groups |
| Outcomes | 6a | Completely defined prespecified assessments or measurements to address each pilot trial objective specified in 2b, including how and when they were assessed | 6 / Outcomes and data definiton |
|  | 6b | Any changes to pilot trial assessments or measurements after the pilot trial commenced, with reasons | N/A |
|  | 6c | If applicable, prespecified criteria used to judge whether, or how, to proceed with future definitive trial | N/A |
| Sample size | 7a | Rationale for numbers in the pilot trial | 6 / Sample size |
|  | 7b | When applicable, explanation of any interim analyses and stopping guidelines | N/A |
| Randomisation: |  |  |  |
| Sequence  generation | 8a | Method used to generate the random allocation sequence | 4 / Randomization |
|  | 8b | Type of randomisation(s); details of any restriction (such as blocking and block size) | 4 / Randomization |
| Allocation  concealment  mechanism | 9 | Mechanism used to implement the random allocation sequence (such as sequentially numbered containers), describing any steps taken to conceal the sequence until interventions were assigned | 4 / Randomization |
| Implementation | 10 | Who generated the random allocation sequence, who enrolled participants, and who assigned participants to interventions | 4 / Randomization |
| Blinding | 11a | If done, who was blinded after assignment to interventions (for example, participants, care providers, those assessing outcomes) and how | 5 / Treatment groups |
|  | 11b | If relevant, description of the similarity of interventions | N/A |
| Statistical methods | 12 | Methods used to address each pilot trial objective whether qualitative or quantitative | 7 / Data analysis |
| Results | | | |
| Participant flow (a diagram is strongly recommended) | 13a | For each group, the numbers of participants who were approached and/or assessed for eligibility, randomly assigned, received intended treatment, and were assessed for each objective | Figure 1 |
|  | 13b | For each group, losses and exclusions after randomisation, together with reasons | Figure 1 |
| Recruitment | 14a | Dates defining the periods of recruitment and follow-up | 8 / Results |
|  | 14b | Why the pilot trial ended or was stopped | N/A |
| Baseline data | 15 | A table showing baseline demographic and clinical characteristics for each group | Table 1 |
| Numbers analysed | 16 | For each objective, number of participants (denominator) included in each analysis. If relevant, these numbers  should be by randomised group | Results |
| Outcomes and estimation | 17 | For each objective, results including expressions of uncertainty (such as 95% confidence interval) for any  estimates. If relevant, these results should be by randomised group | Results, Figure 2,3  Table 2,3 |
| Ancillary analyses | 18 | Results of any other analyses performed that could be used to inform the future definitive trial | N/A |
| Harms | 19 | All important harms or unintended effects in each group (for specific guidance see CONSORT for harms) | Table 3 |
|  | 19a | If relevant, other important unintended consequences | N/A |
| Discussion | | | |
| Limitations | 20 | Pilot trial limitations, addressing sources of potential bias and remaining uncertainty about feasibility | 16 |
| Generalisability | 21 | Generalisability (applicability) of pilot trial methods and findings to future definitive trial and other studies | 16 |
| Interpretation | 22 | Interpretation consistent with pilot trial objectives and findings, balancing potential benefits and harms, and  considering other relevant evidence | 15 |
|  | 22a | Implications for progression from pilot to future definitive trial, including any proposed amendments | 16 |
| Other information | | |  |
| Registration | 23 | Registration number for pilot trial and name of trial registry | 2 |
| Protocol | 24 | Where the pilot trial protocol can be accessed, if available | N/A |
| Funding | 25 | Sources of funding and other support (such as supply of drugs), role of funders | 17 / Funding sources |
|  | 26 | Ethical approval or approval by research review committee, confirmed with reference number | 2 |

# Supplement Table 1 – Inclusion and Exclusion Criteria

| **Inclusion** | **Exclusion** |
| --- | --- |
| ≥18 years | Known pregnancy |
| ALS interventions complete   - Endotracheal intubation - i.v. access - ALS drugs applied | Prisoners |
| Anticipated resuscitation duration >10 minutes | Traumatic cardiac arrest |
|  | Known esophageal disease |
|  | Known esophageal operation |
|  | Suspected gastrointestinal bleeding |
|  | DNR order |

# Supplement Table 2 – Primary and secondary endpoints

| **Primary Endpoints** | **Secondary endpoints** |
| --- | --- |
| Hands off time | Any-ROSC |
| Chest compression fraction | ROSC at hospital admission |
|  | Survival to hospital discharge |
|  | Neurological status at hospital admission |
|  | Changes in end-tidal CO_2_ |
|  | TEE Image quality |
|  | Complications   - Extubation - Bleeding - Teeth damage - Esophageal perforation - Tracheal injuries |
|  | TEE image findings   - Potential cause of OHCA - eCPR guidance and visualization of guidewire & cannula |

# Supplement Table 3 – Sensitivity analysis CCF before decision to perform eCPR

|  | CCF [%], mean | CCF [%], SD | 95% CI |
| --- | --- | --- | --- |
| **TEE, n=15** | 95.6 | 2.2 | 94.3 – 96.8 |
| **Standard ALS, n=17** | 91.3 | 3.4 | 89.5 – 93.0 |
| **Overall, n=32** | 93.3 | 3.6 | 92.0 – 94.6 |

Abbreviations: CCF = Chest compression fraction; SD = standard deviation; CI = confidence interval; TEE = Trans esophageal echocardiography; ALS = advanced life support

# Supplement Table 4 – Rates of Hands off times >10s before and after the study team arrives

| **Study Team on Scene** | **Study Group** | **Hands off ≤10s (.00)** | **Hands off >10s (1.00)** | **Total** | **Rate >10s (%)** |
| --- | --- | --- | --- | --- | --- |
| **No** | Intervention | 125 | 17 | 142 | 11.97% |
| **No** | Control | 147 | 12 | 159 | 7.55% |
| **Yes** | Intervention | 46 | 13 | 59 | 22.03% |
| **Yes** | Control | 26 | 5 | 31 | 16.13% |

| **Context** | **Intervention (%)** | **Control (%)** | **Difference (Interv – Control)** | **p-value** |
| --- | --- | --- | --- | --- |
| **Study Team on Scene** | 12.0 | 7.6 | +4.4 | 0.24 |
| **Study Team on Scene** | 22.0 | 16.1 | +5.9 | 0.59 |
