# Supplementary material for: Prehospital transesophageal echocardiography versus conventional advanced life support in out-of-hospital cardiac arrest (PHTEE–OHCA) – a randomized controlled pilot study
Source: Crit Care. 2026 Jan 2;30:45. doi: 10.1186/s13054-025-05805-w (PMC12849066; doi:10.1186/s13054-025-05805-w)

Prehospital transesophageal echocardiography in out-of-hospital cardiac arrest (PHTEE – OHCA) – a randomized controlled pilot study

The study

Screening:

249 adults with out-of-hospital cardiac arrest

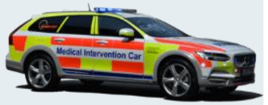

35 randomized on scene

32 were analysed in intention-to-treat

Intervention:
ALS + TEE (n=15)

Control:
ALS (n=17)

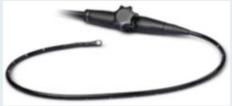

Primary Outcome:
Hands-off time and chest compression fraction

Implications

TEE significantly improved chest compression fraction and end-tidal CO2 levels for out-of-hospital cardiac arrest patients, along with essential diagnostic insights and helpful procedural guidance for optimal care.

Findings

Patients in the TEE group had similar hands-off time, improved chest compression fraction, and an increase in etCO2. TEE revealed an incorrect area of maximal compression or inadequate depth in over one-third of the cases.

Hands-off time

ALS + TEE: 4 sec* (3 to 8)

ALS: 4 sec* (3 to 6)

Chest compression fraction

Mean difference
4.6% (95% CI 2.5 to 6.7)

ALS + TEE: 96.2%

ALS: 91.6%

End tidal CO2

p<0.001

ALS + TEE: 30

ALS: 24

*Difference of medians 0.0 sec 95% CI 0.0-1.0, p=0.047

Area of maximal compression not over LV (23%) and inadequate depth (14%)

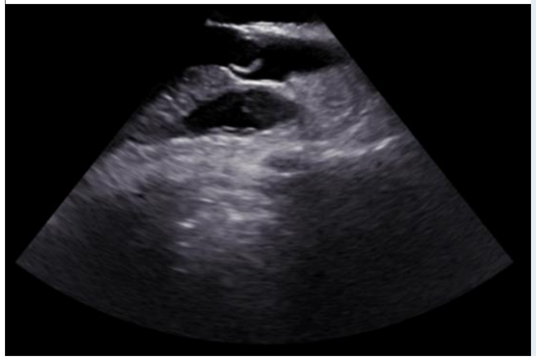

eCPR Guidance with cannula and guidewire

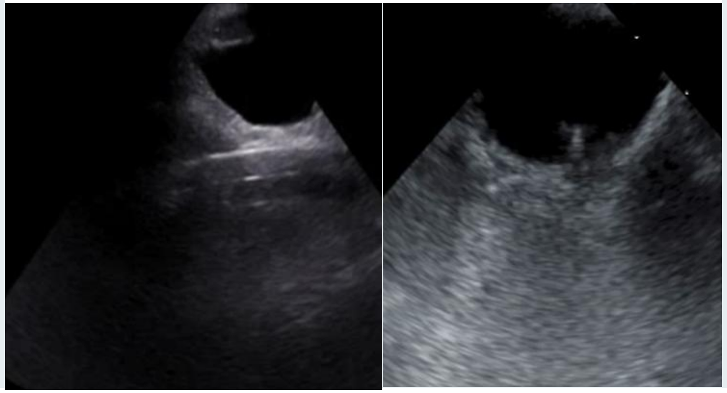

Pericardial effusion (23%)

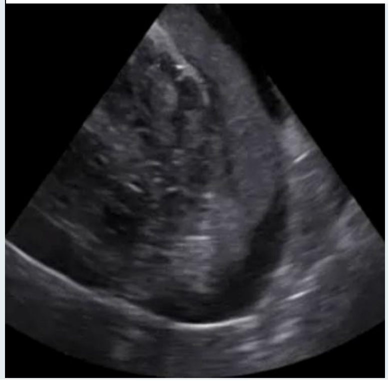

Supplement: Supplementary file 5 — Supplementary Material 5 [file 13054_2025_5805_MOESM5_ESM.pdf]
